# *Macrocystis pyrifera* Lipids Reduce Cytokine-Induced Pro-Inflammatory Signalling and Barrier Dysfunction in Human Keratinocyte Models

**DOI:** 10.3390/ijms242216383

**Published:** 2023-11-16

**Authors:** Jamie M. L. Kok, Georgina C. Dowd, Jaydee D. Cabral, Lyn M. Wise

**Affiliations:** 1 Department of Pharmacology and Toxicology, School of Biomedical Sciences, University of Otago, Dunedin 9054, New Zealand; jamie.kok@qut.edu.au; 2 The New Zealand Institute for Plant and Food Research Limited, Nelson 7043, New Zealand; georgina.dowd@plantandfood.co.nz; 3 Department of Microbiology and Immunology, School of Biomedical Sciences, University of Otago, Dunedin 9054, New Zealand; jaydee.cabral@otago.ac.nz

**Keywords:** atopic dermatitis, brown seaweed, *Macrocystis pyrifera*, lipid, keratinocyte, epidermal barrier, inflammation, cytokine, chemokine, tight junction

## Abstract

Atopic dermatitis is a chronic condition where epidermal barrier dysfunction and cytokine production by infiltrating immune cells exacerbate skin inflammation and damage. A total lipid extract from *Macrocystis pyrifera*, a brown seaweed, was previously reported to suppress inflammatory responses in monocytes. Here, treatment of human HaCaT keratinocytes with *M. pyrifera* lipids inhibited tumour necrosis factor (TNF)-α induced TNF receptor-associated factor 2 and monocyte chemoattractant protein (MCP)-1 protein production. HaCaT cells stimulated with TNF-α, interleukin (IL)-4, and IL-13 showed loss of claudin-1 tight junctions, but little improvement was observed following lipid pre-treatment. Three-dimensional cultures of HaCaT cells differentiated at the air–liquid interface showed increased MCP-1 production, loss of claudin-1 tight junctions, and trans-epidermal leakage with TNF-α, IL-4, and IL-13 stimulation, with all parameters reduced by lipid pre-treatment. These findings suggest that *M. pyrifera* lipids have anti-inflammatory and barrier-protective effects on keratinocytes, which may be beneficial for the treatment of atopic dermatitis or other skin conditions.

## 1. Introduction

Atopic dermatitis is a common inflammatory skin condition that is prevalent in children (15–30%) and to a lesser extent in adults (1–3%) [1]. Atopic dermatitis is characterised by a damaged skin barrier, recurrent infection, and a transition from a T helper (Th)2 to Th1 immune response during disease progression [2,3].

The epidermis of the skin is made of stratified keratinocytes that form a replicative basal layer and numerous differentiated suprabasal layers [4]. Keratinocytes function as the main structural and barrier components of the epidermis [5] and play a critical role in the initiation and maintenance of skin inflammatory and wound healing responses [6]. Keratin 5 and keratin 14 are expressed exclusively in basal keratinocytes, whereas keratin 1, keratin 10, involucrin, and filaggrin are expressed upon their differentiation [4,5]. In the most superficial epidermal layer, flattened keratinocytes called corneocytes are embedded in a lipid matrix consisting of ceramides, cholesterol, and free fatty acids [7]. Tight junctions between keratinocytes are critical to maintaining the epidermal barrier [8,9]. Claudin-1, a tight junction protein that predominates in the skin [10], regulates keratinocyte proliferation and differentiation [11], with amounts inversely correlating to the extent of epidermal barrier leakage [12,13,14]. Disruption to this epidermal barrier contributes to the development and severity of atopic dermatitis [15], with changes in lipid content correlating to trans-epidermal water loss and colonisation by *Staphylococcus aureus* in atopic children and dogs [16,17,18].

The acute and chronic phases of atopic dermatitis are characterised by Th2 and Th1 lymphocyte responses, respectively [19,20]. Th cells maintain atopic immune responses in the skin through the secretion of specific effector cytokines [3,20]. For example, tumour necrosis factor (TNF)-α and interferon (IFN)-γ are produced by Th1 cells, while Th2 cells produce interleukin (IL)-4, IL-5, and IL-13. Th2 cytokines are considered the most potent activators of keratinocytes, modulating tight junctions which control epidermal barrier function through the induction of signal transducer and activator of transcription (STAT) signalling [21,22]. Th1 cytokines also activate nuclear factor kappa-light-chain-enhancer of activated B cell (NF-kB) signalling in keratinocytes, increasing their production of pro-inflammatory chemokines such as monocyte chemoattractant protein (MCP)-1, which exacerbates skin inflammation through recruitment of neutrophils and monocytes from the blood [23,24,25].

Treatments for atopic dermatitis fall into two categories: lipid-based emulsions that coat, protect, and hydrate the skin, and synthetic drugs that suppress inflammatory signalling within the skin [26]. Topical corticosteroids, such as hydrocortisone, mediate their anti-inflammatory effects by binding with the glucocorticoid receptor suppressing NF-kB-mediated transcription of pro-inflammatory mediators. Emollients containing plant-derived lipids provide an artificial barrier against trans-epidermal water loss and environmental triggers [27]. A systemic review supported that emollients alleviate atopic dermatitis symptoms, particularly when combined with corticosteroids [28]. However, issues with efficacy, tolerability, and patient compliance with these treatment classes have led to the search for therapeutic alternatives [26,29].

Human keratinocyte and epidermal models are commonly used to assess the utility of topical therapeutics prior to testing in animal models [30,31,32]. In general, keratinocytes isolated from human skin maintain a basal phenotype in the presence of low calcium concentrations and differentiate when exposed to high calcium (>0.1 mM) [33]. But in vitro experimentation with primary keratinocytes has limitations, including a short lifespan and requirement for growth factor supplementation [34]. As a result, immortalised human keratinocyte cell lines are often used for in vitro research owing to their capacity to avoid senescence and survive indefinitely in culture. HaCaT cells, a spontaneously transformed aneuploid immortal keratinocyte cell line derived from adult human skin, closely mimic the phenotype of primary human keratinocytes [33]. Stimulation of HaCaT cells with IFN-γ, TNF-α, and/or IL-4 alter the expression of pro-inflammatory mediators and differentiation proteins, as reported in skin lesions of patients with atopic dermatitis [35]. HaCaT cells also express tight junction proteins, such as claudin 1, similarly to human neonatal and adult keratinocytes [36]. After culture at the air–liquid interface, HaCaT cells also form a squamous epithelium that expresses most differentiation markers [37]. Additionally, when transplanted onto the subcutaneous tissue of nude mice, HaCaT cells developed a normal epidermal architecture [38]. HaCaT cells have been employed to investigate epidermal biology, skin pathologies, and topical therapies [39,40,41].

Seaweed-derived compounds have been identified that modulate immunological processes involved in atopic dermatitis [42,43]. Anti-inflammatory activity in monocytes has been reported with lipids from *Macrocystis pyrifera* [44], *Undaria pinnatifida* and *Undaria linza* [45], phlorotannins from *Ecklonia cava* [46], and sulphated polysaccharides from *Sargassum hemiphyllum* and *Sargassum horneri* [47,48]. Further, *Capsosiphone fulvescens* preparation reduced pro-inflammatory cytokine levels in TNF-α- and IFN-γ-stimulated HaCaT keratinocytes and infiltration of inflammatory cells in a mouse model of *Dermatophagoides farinae*-induced atopic dermatitis [49]. These compounds have been shown to suppress inflammation through reduction in the expression, phosphorylation, or nuclear translocation of NF-κB, mitogen-activated protein kinase (MAPK), and STAT signalling proteins [44,47,48,49].

Additionally, seaweed components have been reported to preserve epithelial barrier function in a manner that may be useful for the treatment of atopic dermatitis. An aqueous extract from *Laminaia japonica* reduced lipoteichoic acid-stimulated intestinal permeability in polarised Caco-2 epithelial cell monolayers by restoring expression of the tight junction protein, occludin [50]. In addition, a sulphated polysaccharide from *Cladosiphon okamuranus Tokida* restored claudin-1 and claudin-2 protein abundance and prevented H_2_O_2_-induced permeability in the same intestinal model [51]. As well, an extract containing a mixture of fucoidan and fucoxanthin from *S. hemiphyllum* enhanced occludin, claudin-1, and claudin-2 mRNA expression in Caco-2 cells challenged with lipopolysaccharide (LPS) [52]. Further, lipid-rich extracts from *Himantothallus grandifolius*, *Plocamium cartilagineum*, *Phaeurus antarcticus*, and *Kallymenia antarctica* restored barrier integrity to Caco-2 and HaCaT monolayers exposed to LPS or ultraviolet (UV)-B light, respectively [53].

Lipid emulsions are used therapeutically to provide a protective skin barrier, while seaweed lipids have been shown to inhibit inflammation and damage induced by Th1 cytokines, pathogen-derived toll-like receptor agonists, and UV-B light. However, little is known as to whether lipid emulsions or seaweed extracts modulate skin inflammation and barrier dysfunction induced by Th2 cytokines. Therefore, this study investigated the effects of lipids extracted from the brown seaweed, *M. pyrifera*, on Th1 and Th2 cytokine-mediated inflammation and tight junction dysfunction in HaCaT cell monolayers and 3D epidermal constructs. This study aimed to determine if seaweed lipids could offer any therapeutic benefit for individuals suffering from atopic dermatitis.

## 2. Results

### 2.1. M. pyrifera Lipids Inhibit Pro-Inflammatory Signalling in Keratinocyte Monolayers Stimulated by the Th1 Cytokine TNF-α

**Figure 1:**
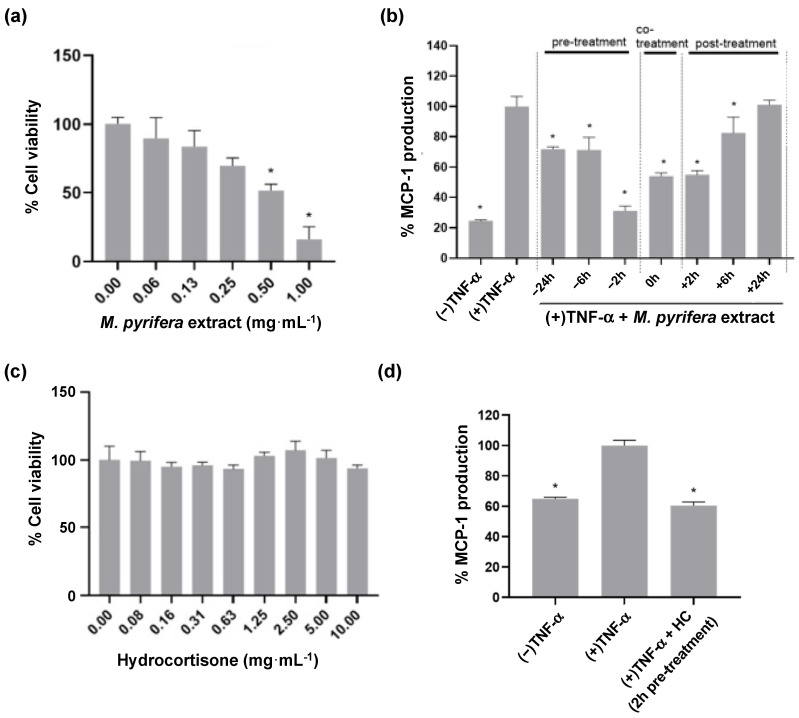
*Macrocystis pyrifera* lipid pre-treatment reduces MCP-1 production in TNF-α-stimulated HaCaT cell monolayers to a similar extent as hydrocortisone. Cells were incubated for 24 h with (**a**) *M. pyrifera* extract or (**c**) hydrocortisone (HC), with biomass assessed by crystal violet staining. Values are expressed relative to the vehicle-only control (0 mg·mL^−1^). Cells were stimulated with TNF-α (20 ng·mL^−1^) with or without (**b**) pre-, co-, or post-treatment with extract (0.13 mg·mL^−1^) or (**d**) 2 h pre-treatment with HC (10 mg·mL^−1^). After 24 h incubation, conditioned medium was collected and assayed for MCP-1 production by ELISA. (−) represents unstimulated cells. (+) represents cells stimulated with TNF-α. Values are expressed as percentages of the (**a**,**c**) vehicle or (**b**,**d**) (+) controls and presented as means ± SD (*n* = 3), with those that differ significantly from those controls identified by one-way ANOVA followed by a Dunnett’s test (* *p* ≤ 0.05).

HaCaT cell monolayers were treated with a total lipid extract from *M. pyrifera* prepared previously [44], with cytotoxicity assessed using a crystal violet assay. At concentrations of ≥0.5 mg·mL^−1^, cells showed reduced viability relative to the vehicle-only control (48–84%, Figure 1a, *p* ≤ 0.05). For subsequent testing, an extract concentration of 0.13 mg·mL^−1^ was used, as this suppressed inflammatory signalling in monocytes [44].

HaCaT cell monolayers were stimulated with Th1 cytokine TNF-α and treated with *M. pyrifera* extract, with effects on the production of the chemokine MCP-1 determined using an enzyme-linked immunosorbent assay (ELISA). The concentration of TNF-α utilised was guided by prior reports [33,35,49,54,55]. The TNF-α-induced increase in MCP-1 production was significantly reduced by 2, 6, and 24 h pre-treatment and co-treatment, and 2 and 6 h post-treatment (Figure 1b, *p* ≤ 0.05), with 2 h extract pre-treatment reducing levels closest to that of unstimulated cells. Thus, for subsequent analyses, treatments were applied 2 h before cytokine stimulation. Hydrocortisone was investigated as a positive control due to its application as a topical treatment for skin inflammation. Hydrocortisone was non-toxic to HaCaT cell monolayers at concentrations ≤ 10 mg·mL^−1^ (Figure 1c). Hydrocortisone pre-treatment at the non-toxic concentration of 10 mg·mL^−1^ significantly suppressed MCP-1 production stimulated by TNF-α to below that of unstimulated cells (Figure 1d, *p* ≤ 0.05). These findings indicate that *M. pyrifera* lipids reduce TNF-α-induced chemokine production in keratinocyte monolayers to a similar extent as hydrocortisone.

**Figure 2:**
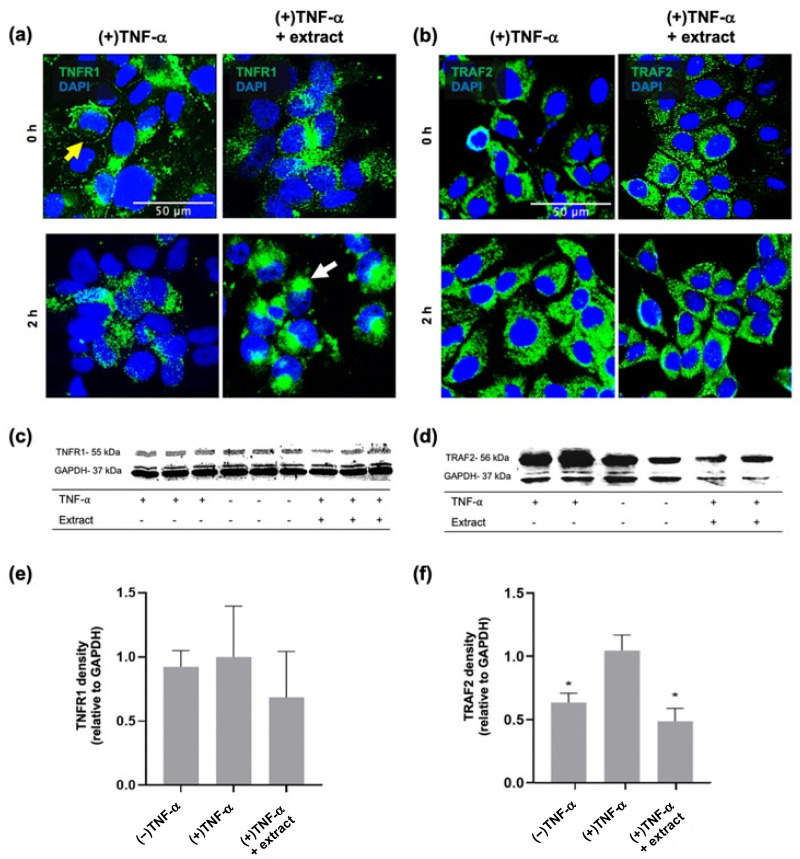
*Macrocystis pyrifera* lipid pre-treatment modulates TNFR1 localisation and TRAF2 production in TNF-α-stimulated HaCaT cell monolayers. HaCaT cells were incubated with TNF-α (20 ng·mL^−1^) with or without 2 h pre-treatment with *M. pyrifera* extract (0.13 mg·mL^−1^). After 0 and 2 h stimulation with TNF-α, cells were (**a**) fixed for extracellular staining or (**b**) fixed and permeabilised for intracellular staining, using rabbit (**a**) anti-TNFR1 or (**b**) anti-TRAF2 and anti-rabbit Alexa Fluor^®^488 antibodies (green). Nuclei were stained using DAPI (blue). Representative images are shown, with yellow and white arrows indicating cell junction and pole localisation, respectively. Scale bar, 50 μm. After 2 or 4 h stimulation with TNF-α, Western blot analyses were performed on cell lysates using rabbit anti-GAPDH, (**c**) anti-TNFR1, or (**d**) anti-TRAF2, and anti-rabbit HRP antibodies. Band densities were determined from Western blots detecting (**e**) TNFR1 and (**f**) TRAF2. (−) represents unstimulated cells. (+) represents cells stimulated with TNF-α. Values are expressed relative to the (+) control and loading control (GAPDH) and presented as mean ± SD (*n* = 3), with those that differ significantly from the (+) control identified by one-way ANOVA followed by a Dunnett’s test (* *p* ≤ 0.05).

The main cell surface receptor responsible for TNF-*α* signalling within keratinocytes is TNF receptor 1 (TNFR1), which upon ligand binding is translocated to lipid rafts where its intracellular domain interacts with adaptor proteins that recruit TNF receptor associated factor 2 (TRAF2), leading to downstream activation of NF-κB and its transcription of pro-inflammatory cytokines [23,25]. Immunohistochemistry was therefore used to assess TNFR1 signalling in the HaCaT cell monolayer after stimulation with TNF-α and treatment with *M. pyrifera* extract. Cell surface staining for TNFR1 was localised at the junction of unstimulated cells, with punctate staining spread across cells after 2 h stimulation with TNF-α (Figure 2a). TNFR1 staining at cell–cell junctions was also observed after 2 h pre-treatment with the extract; however, after TNF-α stimulation for 2 h, the staining appeared to redistribute to cell poles (Figure 2a). Intracellular staining for TRAF2 was observed near the nuclear membrane of unstimulated cells, with staining increasing and redistributing throughout TNF-α-stimulated cells within 2 h (Figure 2b). In the extract pre-treated cells, TRAF2 staining was distributed throughout the cell, but the staining intensity appeared to be reduced relative to unstimulated and TNF-α-stimulated cells (Figure 2b).

Western blot analyses were used to quantify TNFR1 and TRAF2 abundance in cytokine-stimulated and extract-treated monolayers. Bands were observed as 55 and 56 kDa corresponding to TNFR1 and TRAF2, respectively (Figure 2c,d). Two bands of 36 and 38 kDa were observed for the loading control, glyceraldehyde 3-phosphate dehydrogenase (GAPDH), which may reflect post-translational modifications [56,57]. Differences were noted in loading control density across samples, but these were not consistent across the TNFR1 and TRAF2 Western blots (Figure 2c,d). When normalised to the loading control bands, no changes in TNFR1 abundance were detected (Figure 2e); however, the increase in TRAF2 density with TNF-α stimulation was suppressed by extract pre-treatment (Figure 2f, *p* ≤ 0.05). These findings suggest that *M. pyrifera* lipids may limit MCP-1 production in keratinocyte monolayers by modulating TNFR1 localisation and TRAF2 production.

HaCaT cell monolayers were treated with a fresh *M. pyrifera* total lipid extraction, with cytotoxicity reassessed by crystal violet staining. Similar to the first batch of extract, at concentrations of ≥0.63 mg·mL^−1^, cell viability was reduced (Figure S1a, *p* ≤ 0.05). TNF-α-stimulated HaCaT cell monolayers were pre-treated with this extract (at an equivalent concentration of 0.16 mg·mL^−1^), with MCP-1 production reassessed by ELISA. MCP-1 production was suppressed to levels observed in unstimulated cells (Figure S1b, *p* ≤ 0.05).

These findings indicate that the *M. pyrifera* lipid extracts were equivalent in activity and that an extract concentration of 0.16 mg·mL^−1^ was suitable for subsequent testing.

### 2.2. M. pyrifera Lipids Do Not Prevent Disruption of Claudin-1 Tight Junctions in Keratinocyte Monolayers Stimulated by Th1 and Th2 Cytokines

**Figure 3:**
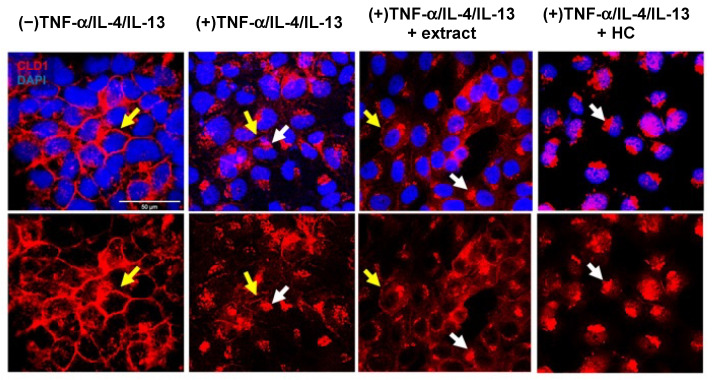
*Macrocystis pyrifera* lipid pre-treatment does not prevent the loss of claudin-1 tight junctions in cytokine-stimulated HaCaT cell monolayers. Cells were incubated for 24 h with TNF-α (20 ng·mL^−1^), IL-4 (50 ng·mL^−1^), and IL-13 (50 ng·mL^−1^), with or without 2 h pre-treatment with *M. pyrifera* extract (0.16 mg·mL^−1^) or hydrocortisone (HC, 10 mg·mL^−1^). Cells were fixed then tight junctions were stained using rabbit anti-claudin-1 (CLD1) and anti-rabbit Alexa Fluor^®^594 antibodies (red), with nuclei stained using DAPI (blue). Representative images are shown, with yellow and white arrows indicating intact and disrupted tight junctions, respectively. (−) represents unstimulated cells. (+) represents cells stimulated with cytokines. Scale bar, 50 µm.

HaCaT cell monolayers were stimulated with the Th1 cytokine TNF-α or the Th2 cytokines IL-4 and IL-13, with immunocytochemistry used to determine their effects on claudin-1 tight junctions. The concentrations of TNF-α, IL-4, and IL-13 utilised were guided by prior reports [35,54,55]. In unstimulated monolayers, claudin-1 staining appeared as a continuous and distinct line at cell–cell junctions (Figure 3 and Figure S2). In TNF-α-stimulated monolayers, claudin-1 remained at the cell–cell junctions, although increased content was observed within the cell (Figure S2). Following IL-4 and IL-13 stimulation, claudin-1 staining was observed at cell–cell junctions, but in less cells than the number observed for unstimulated monolayers (Figure S2). Greater disruption of claudin-1 staining from cell–cell junctions was observed in monolayers stimulated concurrently with TNF-α, IL-4, and IL-13 (Figure 3 and Figure S2).

Claudin-1 tight junctions in TNF-α-, IL-4-, and IL-13-stimulated HaCaT cell monolayers were assessed after pre-treatment with the fresh *M. pyrifera* extract. A similar claudin-1 staining pattern was observed relative to cytokine-stimulated cells but with a greater number of cells observed with light staining at cell–cell junctions (Figure 3). This was not observed in cytokine-stimulated cells following hydrocortisone pre-treatment, where every cell showed complete disruption of claudin-1 tight junctions (Figure 3).

The abundance of claudin-1 in HaCaT cell monolayers was also quantified using Western blotting. Claudin-1 was observed as a single band at ≈20 kDa (Figure S3a), while the loading control β-tubulin was identified at ≈55 kDa. Neither cytokine stimulation nor extract pre-treatment altered the claudin-1 band density relative to unstimulated cells (Figure S3b, *p* > 0.05).

### 2.3. M. pyrifera Lipids, in Part, Reduce Trans-Epidermal Leakage, Chemokine Production, and Claudin-1 Tight Junction Disruption in 3D Epidermal Constructs Stimulated by Th1 and Th2 Cytokines

**Figure 4:**
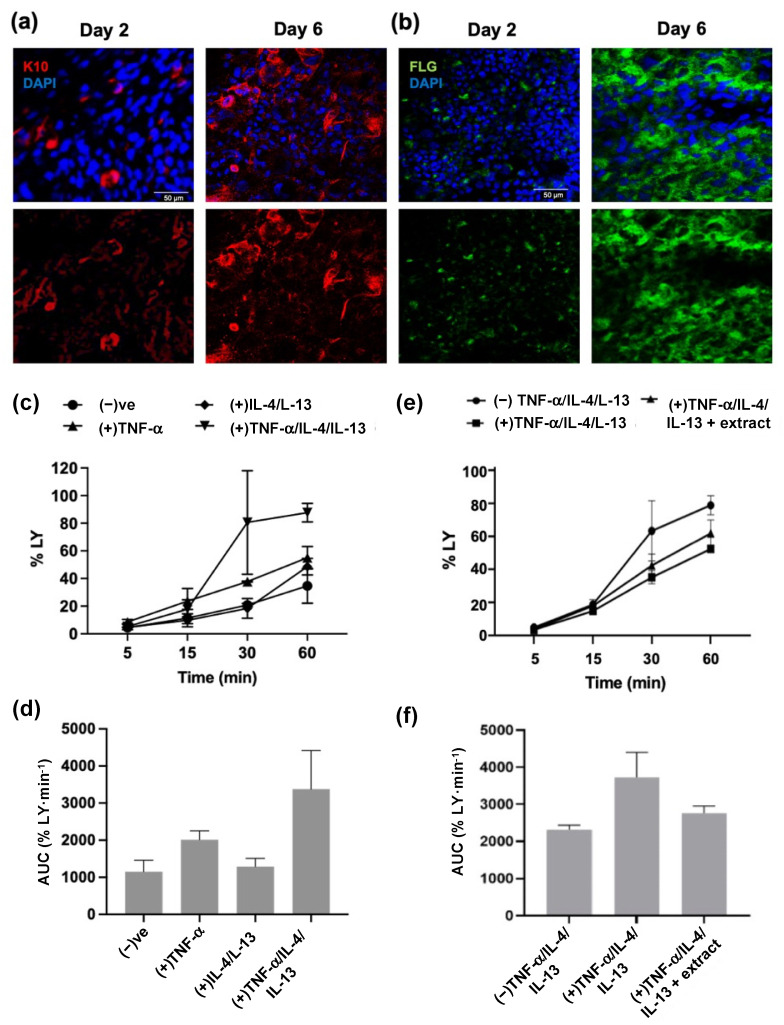
*Macrocystis pyrifera* lipid pre-treatment reduces trans-epidermal leakage in cytokine-stimulated 3D epidermal constructs. HaCaT cells were cultured for 2 days, immersed in proliferation media, followed by 6 days of culture at the air–liquid interface on differentiation media. At day 2 and 6 of differentiation, the constructs were whole-mount stained with rabbit (**a**) anti-keratin 10 (K10) or (**b**) anti-filaggrin (FLG) and anti-rabbit Alexa Fluor^®^594 (red) or Alexa Fluor^®^488 (green) antibodies, respectively, and nuclei stained with DAPI (blue). Representative optical slices from confocal Z-stacks (Figure S4) are shown. Scale bar, 50 µm. (**c**) 3D constructs were stimulated for 24 h with TNF-α (20 ng·mL^−1^) and/or IL-4 (50 ng·mL^−1^) and IL-13 (50 ng·mL^−1^). Lucifer yellow (LY; 100 µg·mL^−1^) was applied topically, with cumulative levels permeating through 3D cell cultures determined using a fluorometer. Values are expressed as percentages of the total LY. (**d**) The area under the curve (AUC) calculated for each stimulation in (**c**). (**e**) 3D constructs were stimulated for 24 h with TNF-α (20 ng·mL^−1^), IL-4 (50 ng·mL^−1^), and IL-13 (50 ng·mL^−1^), with or without 2 h pre-treatment with *M. pyrifera* extract (0.16 mg·mL^−1^). LY was applied and quantified as described in (**c**). (**f**) The AUC calculated for each stimulation/treatment in (**e**). (−) represents unstimulated 3D constructs. (+) represents 3D cell constructs stimulated with cytokines. Values are expressed as the means ± SD (*n* = 2 (**c**,**d**); *n* = 4 (**e**,**f**)). No significant differences between means were identified in (**f**) using one-way ANOVA (*p* > 0.05).

To more accurately mimic the human epidermis and facilitate measures of barrier functionality, HaCaT cells were cultured in Transwell^®^ inserts for 2 days, then lifted to the air–liquid interface and cultured with differentiation stimuli for 6 days [53,58,59]. The differentiation state of the constructs was evaluated at day 2 and 6 of the differentiation protocol using whole-mount immunocytochemistry and confocal microscopy. Optical slices from confocal Z-stacks revealed increased expression of markers of keratinocyte differentiation, namely, keratin-10 and filaggrin, from day 2 to day 6 (Figure 4a,b). Keratin-10 staining was distributed throughout the cell layers at day 6 (Figure S4a), while filaggrin staining was localised to the apical side of the cell layers (Figure S4b). These observations suggest that 3D constructs were produced which could be used to assess barrier function.

HaCaT cell constructs were stimulated with the Th1 cytokine TNF-α or the Th2 cytokines IL-4 and IL-13, individually and in combination, with effects on trans-epidermal leakage assessed using a dye exclusion assay. The TNF-α, IL-4, and IL-13 concentrations were as used previously, as they were consistent with previous studies in 3D skin equivalents [55]. A dye exclusion assay was used to assess barrier function as prior reports indicated HaCaT cells do not support measures of trans-epithelial electrical resistance [53,60] but do limit permeation of topically applied fluorescent dyes such as lucifer yellow [53,55,61,62]. With unstimulated controls, ≈35% of the lucifer yellow passed from the apical to the basal side of the 3D constructs within 60 min (Figure 4c). While constructs stimulated with IL-4 and IL-13 showed negligible change in dye leakage relative to that of unstimulated controls, TNF-α-stimulated constructs showed a cumulative increase in lucifer yellow leakage, with ≈55% passing through the construct after 60 min (Figure 4c). The greatest increase in lucifer yellow leakage was, however, observed following stimulation with TNF-α, IL-4, and IL-13, with ≈80% passing through the construct after 60 min (Figure 4c), culminating in a ≈3.3-fold increase in the rate of dye leakage than unstimulated constructs (Figure 4d). In subsequent experiments, TNF-α, IL-4, and IL-13 cytokines were used in combination to induce epidermal barrier dysfunction.

HaCaT cell constructs were pre-treated with the extract for 2 h, then stimulated with TNF-α, IL-4, and IL-13, with trans-epidermal leakage determined using the dye exclusion assay. 3D constructs stimulated with cytokines showed ≈78% lucifer yellow dye leakage at 60 min, while leakage in the extract pre-treated constructs was similar to that from the unstimulated controls (58% versus 55%, respectively, Figure 4e). The rate of dye leakage increased ≈1.6-fold following cytokine-stimulation (Figure 4f) but remained similar to that of unstimulated constructs following extract pre-treatment. These findings suggest that the *M. pyrifera* extract may limit cytokine-induced trans-epidermal leakage of lucifer yellow dye.

**Figure 5:**
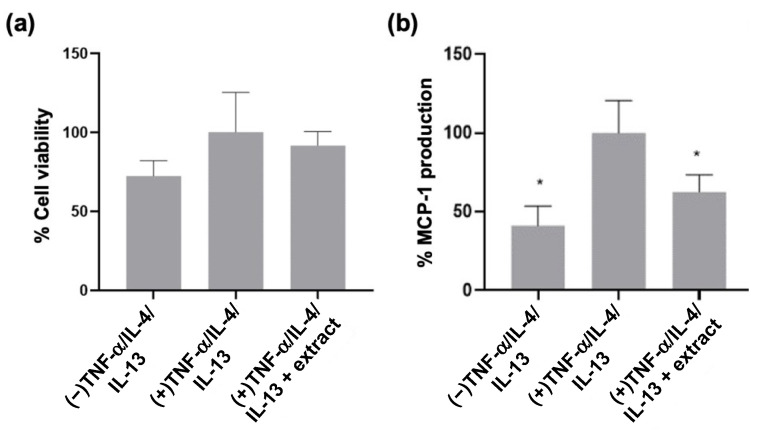
*Macrocystis pyrifera* lipid pre-treatment reduces chemokine production in cytokine-stimulated 3D epidermal constructs. HaCaT cells differentiated at the air–liquid interface were incubated for 24 h with TNF-α (20 ng·mL^−1^), IL-4 (50 ng·mL^−1^), and IL-13 (50 ng·mL^−1^), with or without 2 h pre-treatment with *M. pyrifera* extract (0.16 mg·mL^−1^). Conditioned medium was collected and assayed for (**a**) LDH release or (**b**) MCP-1 production. (−) represents unstimulated 3D constructs. (+) represents 3D constructs stimulated with cytokines. Values are expressed as percentages of the (+) control and presented as means ± SD (*n* = 3), with those that differ significantly from the (+) control identified by one-way ANOVA followed by a Dunnett’s test (* *p* ≤ 0.05).

The viability of the 3D epidermal constructs after cytokine stimulation and extract pre-treatment was assessed using a lactate dehydrogenase (LDH) assay. No significant differences in LDH levels were detected between constructs (Figure 5a). MCP-1 production by the 3D epidermal constructs after cytokine stimulation and extract pre-treatment was assessed by ELISA. Stimulation with TNF-α, IL-4, and IL-13 increased MCP-1 production relative to that of unstimulated constructs, while pre-treatment with *M. pyrifera* extract significantly suppressed this response (Figure 5b, *p* ≤ 0.05). These findings indicate that the *M. pyrifera* extract limits cytokine-induced chemokine production in epidermal constructs without compromising viability.

**Figure 6:**
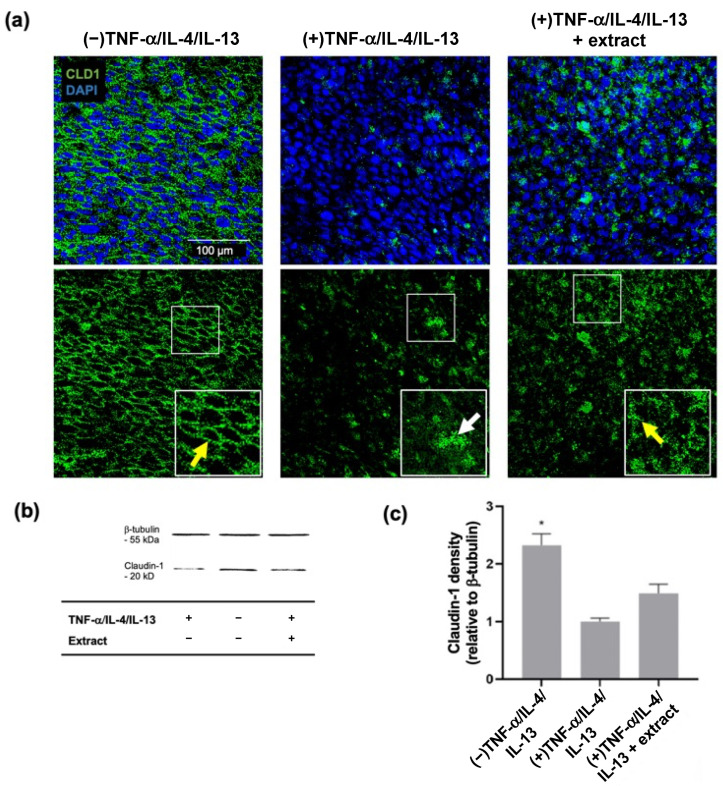
*Macrocystis pyrifera* lipid pre-treatment partially prevents claudin-1 tight junction disruption in cytokine-stimulated 3D epidermal constructs. HaCaT cells differentiated at the air–liquid interface were incubated for 24 h with TNF-α (20 ng·mL^−1^), IL-4 (50 ng·mL^−1^), and IL-13 (50 ng·mL^−1^), with or without 2 h pre-treatment with extract. (**a**) Stimulated 3D constructs were whole-mount stained with anti-claudin-1 (CLD1) and anti-rabbit Alexa Fluor^®^488 (green) antibodies, with nuclei stained with DAPI (blue). Representative optical slices from confocal Z-stacks are shown. Higher magnification inserts (75 × 75 µm) show yellow and white arrows indicating intact and disrupted tight junctions, respectively. Scale bar, 100 µm. (**b**) Western blot analyses were performed on 3D construct lysates using rabbit anti-claudin-1, anti-β-tubulin, and anti-rabbit HRP antibodies. (**c**) Band densities were determined from Western blots detecting claudin-1. (−) represents unstimulated 3D constructs. (+) represents 3D constructs stimulated with cytokines. Values are expressed relative to the (+) control and loading control (β-tubulin) and presented as means ± SD (*n* = 3), with those that differ significantly from the (+) control identified by one-way ANOVA followed by a Dunnett’s test (* *p* ≤ 0.05).

Claudin-1 tight junctions were assessed in TNF-α-, IL-4-, and IL-13-stimulated and *M. pyrifera* extract-pre-treated 3D epidermal constructs using whole-mount immunocytochemistry and confocal microscopy (Figure 6a). Claudin-1 staining was observed throughout the unstimulated constructs, localising at cell–cell boundaries. Following cytokine stimulation, there was a substantial loss of claudin-1 staining, with punctate staining evident across the construct (Figure 6a). Following extract pre-treatment, claudin-1 staining was increased relative to the stimulated constructs, with both punctate and junctional staining observed (Figure 6a).

Western blot analyses were used to quantify claudin-1 abundance in cytokine-stimulated and extract-treated 3D epidermal constructs. Bands corresponding to claudin-1 and β-tubulin were detected for all constructs, at ≈20 and ≈55 kDa, respectively (Figure 6b). Stimulation with cytokines significantly decreased the band density for claudin-1 by ≈58% (Figure 6c, *p* ≤ 0.05), while a partial restoration in claudin-1 protein levels was observed in constructs pre-treated with the extract. These findings suggest that the *M. pyrifera* lipid extract may protect claudin-1 tight junctions in the 3D epidermal constructs from cytokine-induced disruption to a greater extent than that observed in HaCaT cell monolayers.

### 2.4. Fatty Acid Composition of M. pyrifera Lipid Extracts

The fatty acid composition of the *M. pyrifera* extract prepared for this study was evaluated using gas chromatography-mass spectrometry (GC-MS) (Figure S5), with individual fatty acids quantified as percentages of the total (Table S1). In this extract, saturated fatty acids were most abundant, at ≈30%, with palmitic acid being the largest constituent (C16:0, ≈21%). Omega-6 fatty acids represented ≈22%, with the most abundant being arachidonic acid (C20:4n-6, ≈14%) and linoleic acid (C18:2n-6, ≈8%). As oleic acid and α-linolenic acid co-eluted in this analysis (C18:1 + C18:3n-3, ≈30%), the amounts of monounsaturated and omega-3 fatty acids were unable to be quantified. The next most prevalent monounsaturated and omega-3 fatty acids were palmitoleic acid (C16:1, ≈2%) and eicosapentaenoic (C20:5n-3, ≈8%), respectively. When compared to the previously reported extract [44], the total abundance of saturated, monounsaturated, and polyunsaturated fatty acid classes was similar (Table S1), but within class variation was evident. The only fatty acid constituent that was equivalent in abundance between extracts was arachidonic acid (Table S1).

## 3. Discussion

This study provides the first evidence of the protective anti-inflammatory and barrier-enhancing effects of seaweed-derived lipids in keratinocyte models of atopic dermatitis. Treatment with *M. pyrifera* lipids reduced inflammatory signalling and chemokine production in keratinocyte monolayers stimulated by the Th1 cytokine TNF-α. Lipid pre-treatment did not protect keratinocyte monolayers against claudin-1 tight junction loss induced by concurrent stimulation with Th1 and Th2 cytokines, TNF-α, IL-4, and IL-13. Epidermal constructs stimulated with these cytokines showed improved barrier functionality after lipid pre-treatment, with partial reductions in chemokine production, claudin-1 tight junction loss, and trans-epidermal leakage observed.

**Figure 7:**
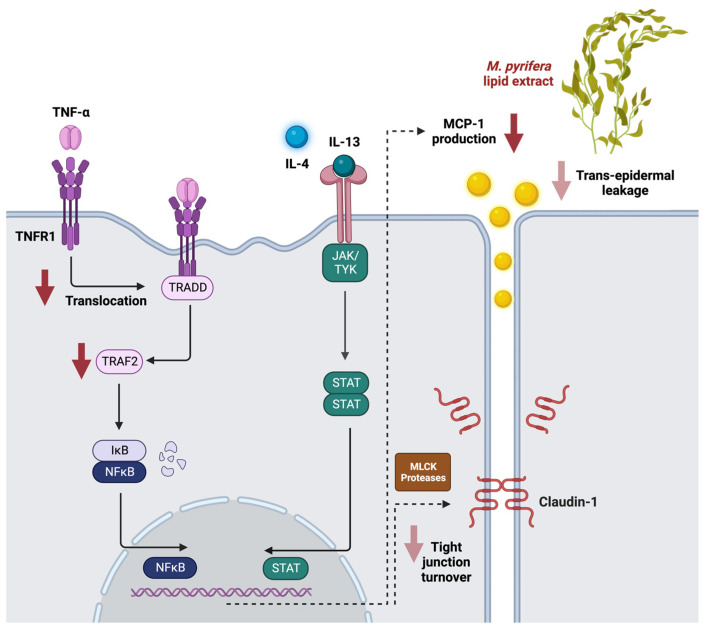
Schematic representation of the anti-inflammatory and barrier protective effects of *M. pyrifera* extract on Th1 and Th2 cytokine-mediated signalling in human keratinocytes. This fatty acid-rich extract reduced MCP-1 production from TNF-α-stimulated HaCaT cells through decreased translocation of TNFR1 and abundance of intracellular TRAF2. The extract also, in part, reduced trans-epidermal leakage and claudin-1 tight junction turnover, primarily mediated by TNF-α. Red arrows depict biological processes and cytokine signalling pathways in keratinocytes suppressed by the *M. pyrifera* lipid extract (arrow density indicates extent of suppression shown in this study). IκB, inhibitor of NFκB; MLCK, myosin light chain kinase; TRADD, tumour necrosis factor receptor type 1-associated death domain. Created with BioRender.com.

The acute and chronic phases of atopic dermatitis are characterised by Th2 and Th1 cell responses, respectively [19,20]. Seaweed lipids have been reported to attenuate pro-inflammatory cytokine production in Th1 cytokine-stimulated keratinocytes. In HaCaT cells stimulated with TNF-α and IFN-γ, an ethanolic extract of *S. horneri* suppressed IL-1β, IL-4, IL-6, IL-13, IFN-γ, and TNF-α [63], while a preparation of *Capsosiphone fulvescens* inhibited TNF-α and IL-6 production [49]. Here, *M. pyrifera* lipids inhibited MCP-1 production in TNF-α-stimulated HaCaT cells to a similar extent as 1% hydrocortisone, a standard treatment for atopic dermatitis. The anti-inflammatory action of *M. pyrifera* lipids was also observed in 3D epidermal constructs stimulated simultaneously with TNF-α, IL-4, and IL-13, a model that more closely replicates atopic skin. Further, lipid pre-treatment altered the cell surface distribution of TNFR1 and inhibited the production of TRAF2, which suggests the lipids may prevent activation of the TNF-α signalling cascade. This is consistent with our prior study, where *M. pyrifera* lipid treatment reduced monocyte production of the cytosolic adapter protein, myeloid differentiation factor 88, preventing NF-kB-mediated inflammatory chemokine production in response to toll-like receptor activation [44]. Together, these findings suggest a common mechanism whereby the anti-inflammatory effects of the *M. pyrifera* lipids are mediated at the cell surface (Figure 7). Indeed, omega-3 fatty acid supplementation has been shown to increase the size and fluidity of lipid microdomains in cell membranes, affecting the assembly of receptors, adapters, and signalling molecules, modulating downstream inflammatory signalling [64,65,66]. Therefore, the *M. pyrifera* lipids could be beneficial for Th1-mediated skin inflammation, offering alternative or combination therapies that could reduce corticosteroid usage and side effects.

Epidermal barrier dysfunction in atopic dermatitis is associated with disruptions to the claudin-1 tight junctions found between keratinocytes [10]. Prior studies suggest that seaweed lipids protect keratinocytes from damage to this barrier. In HaCaT cells exposed to fine dust, an ethanol extract of *S. horneri* increased the abundance of various tight junction proteins, including claudin-1 [67]. Further, various seaweed lipid extracts prevent barrier loss in HaCaT cells induced by UV-B light [53]. In this study, the *M. pyrifera* lipids did not prevent the loss of claudin-1 protein at tight junctions in TNF-α-, IL-4-, and IL-13-stimulated HaCaT cell monolayers, although minor improvements were observed relative to 1% hydrocortisone treatment. In 3D epidermal constructs stimulated simultaneously with these Th1 and Th2 cytokines, treatment with the lipid extract partially restored claudin-1 protein density and tight junction localisation whilst reducing trans-epidermal leakage of lucifer yellow. These findings are in contrast with a previous report, where omega-3 fatty acids increased claudin-1 expression and localisation in intestinal cells treated with 2,4,6-trinitrobenzenesulfonic acid or TNF-α [68,69]. Together, these findings indicate that seaweed lipids may primarily exert barrier protective effects in the context of Th1-mediated inflammation. Indeed, the protective effect of the *M. pyrifera* lipids was greater in the 3D epidermal construct than for the keratinocyte monolayer, where TNF-α, as opposed to IL-4 and IL-13, was the primary mediator of damage. TNF-α-mediated NF-kB signalling directly regulates the expression of tight junction proteins, including various claudins, occludin, and zonula occludens (ZO)-1, and indirectly controls their cellular localisation through the modulation of myosin light chain kinase and intracellular and extracellular protease activity [23,70,71,72]. By contrast, Th2 cytokine-mediated STAT signalling can induce ubiquitination and proteasomal degradation of tight junction proteins, including various claudins and ZO-1 [73]. Omega-3 fatty acids are thought to influence tight junction localisation by altering cell membrane lipid microdomains [74], but the findings here suggest an alternative mechanism via the suppression of the TNFR1-TRAF2-NF-κB signalling pathway (Figure 7). However, investigations are warranted to determine if *M. pyrifera* lipids regulate tight junctions through the turnover pathways differentially regulated by Th1 and Th2 cytokines. Further, these studies should be extended beyond claudin-1 to the constituents of all cellular junctions that control epidermal permeability [8,9].

The anti-inflammatory and barrier-protective effects of the *M. pyrifera* lipid extract should be investigated beyond cultured cells in models that better mimic atopic dermatitis in humans. Although HaCaT cells were successfully used in this study to simulate pathological processes involved in atopic dermatitis, the models used here have limitations. For example, HaCaT cells differ from primary human keratinocytes in their expression of cornified envelope-associated proteins [75], phospholipid content, and cholesterol metabolism [76]. This was evident in the 3D epidermal construct employed here, where only limited layers of keratinocytes were formed, with partial filaggrin coverage and trans-epidermal leakage observed in the absence of cytokine stimulation. It would be interesting in future studies to determine whether treatment with the *M. pyrifera* lipids alters the differentiation status and lipid content or metabolism within the 3D epidermal constructs. Further, in vitro primary human keratinocyte models [30,31], or genetic or chemically induced mouse models of atopic dermatitis [32], could be used to validate these findings using alternative measures of epidermal permeability [8,9] and to assess dosing regimens of topical formulations containing *M. pyrifera* lipids. Gil and colleagues illustrated that a *Capsosiphone fulvescens* extract, which actively suppresses pro-inflammatory cytokine production in stimulated HaCaT cells, inhibited the development of erythema, haemorrhage, edema, scarring, and inflammatory cell infiltration in a NC/Nga mouse model of house dust mite-induced atopic dermatitis [49]. This suggests that the effects of the *M. pyrifera* extract may be replicated in more biologically relevant models.

This study utilised two independent lipid extracts from *M. pyrifera*. The original extract had previously been shown to suppress pro-inflammatory cytokine production in THP-1 monocytes [44], with bioactivity-directed fractionation indicating that myristic acid, palmitoleic acid, and α-linolenic acid mediated this activity without compromising cell viability. Here, both the original and freshly prepared lipid extract suppressed pro-inflammatory chemokine production in HaCaT keratinocytes, at equivalent non-toxic concentrations. This was despite differences being observed in fatty acid composition. In seaweed, the main carriers of fatty acids are complex polar lipids such glycoglycerolipids and phospholipids [77,78]. Esterified polar lipids, such as sulfoquinovosyl diacylglycerols, phosphatidylglycerols, phosphatidylcholines from *Palmaria palmata,* and monogalactosyldiacylglycerols and digalactosyldiacylglycerols from *Chondrus crispus*, showed greater anti-inflammatory properties than isolated polyunsaturated fatty acids [58,79]. This indicates that the polar lipid class, rather than fatty acid composition, may have a greater influence on extract bioactivity. Although further compositional analyses of the *M. pyrifera* extracts would be beneficial, it appears that any differences in their lipid or fatty acid content did not greatly affect their biological activity.

In summary, lipids isolated from *M. pyrifera* reduced inflammatory signalling and barrier dysfunction in keratinocyte monolayers and epidermal constructs, mediated primarily via the Th1 cytokine TNF-α (Figure 7). Further compositional analyses, mechanistic studies, and pre-clinical testing will provide further insight into the reparative effects of *M. pyrifera* lipids for individuals suffering from atopic dermatitis or other skin conditions.

## 4. Materials and Methods

### 4.1. Total Lipid Extracts

Initial experiments utilised a previously reported total lipid extract prepared from *M. pyrifera* samples collected at Wellers Rock, Otago Harbour, New Zealand (45.79° S, 170.71° E), in October 2018 [44]. Due to insufficient stocks, another total lipid extract was prepared from fresh *M. pyrifera* samples for the remaining experiments. Mature thalli were collected during low tide at the subtidal rocky shore from the same location, in September 2020, with a dried and pressed specimen deposited in the University of Otago Herbarium, Dunedin, New Zealand (identifier: OTA72991). This extraction was performed according to methods by Bligh and Dyer [80]. Rinsed and dried seaweed (40 g) was powdered and homogenised (Ultra-Turrax) in chloroform: methanol: water [EMSURE^®^, analytical grade, 400 mL, 1:2:0.8 (*v*/*v*/*v*)]. The macerate was filtered with Whatman filter paper no. 4 and subjected to phase separation with a concentrated saline solution (0.9%, *w*/*v*) overnight at room temperature in a separating funnel. The chloroform layer at the bottom of the flask was collected and evaporated using a vacuum rotary evaporator at 50–55 °C. Extracts were dried under a stream of nitrogen and stored at −20 °C. Extract yields were ≈1.5% and ≈2% of total lipids/gram of dry weight, respectively.

### 4.2. HaCaT Cell Monolayer Culture, Treatment, and Processing

HaCaT cells were cultured in Dulbecco’s modified Eagle’s medium (DMEM, Gibco Inc., Brooklyn, NY, USA), supplemented with 10% heat-inactivated foetal bovine serum (FBS, HyClone Laboratories, San Angelo, TX, USA), 50 U·mL^−1^ penicillin–streptomycin and 60 µg·mL^−1^ kanamycin sulphate (Thermo Fisher Scientific, Waltham, MA, USA). The culture medium contained high calcium concentrations (≥2 mM).

To assess cytotoxicity, HaCaT cell monolayers (1 × 10^5^ cells·mL^−1^) were incubated overnight in 5% CO_2_ at 37 °C with varying concentrations of lipid extract or hydrocortisone (purity ≥ 98%; Sigma, St. Louis, MO, USA) and solubilised in 1% (*v*/*v*) ethanol or DMEM, respectively. To stimulate inflammation or tight junction disruption, HaCaT cell monolayers were stimulated with 20 ng·mL^−1^ TNF-α (purity > 95%, Sino Biological, Beijing, China), with or without IL-4 (purity > 92%, Sino Biological) and IL-13 (purity > 92%, Sino Biological). Unless indicated otherwise, cell monolayers were pre-treated with the extract, hydrocortisone, or vehicle controls (1% ethanol or DMEM) for 2 h, prior to the addition of cytokines.

For chemokine analyses, the conditioned medium was collected by centrifugation after 24 h of incubation with cytokine(s) and then stored at −80 °C. For immunocytochemistry, cells (2.5 × 10^5^ cells·mL^−1^) were seeded on a glass coverslip (8 mm diameter). After 24 h of incubation, cells were fixed with either 4% paraformaldehyde (PFA, Thermo Fisher Scientific) or ice-cold methanol for 5 min, then processed immediately. For Western blot analysis, cells (1 × 10^6^ cells·mL^−1^) were incubated for 24 h, then rinsed with phosphate-buffered saline (PBS, Oxoid, Basingstoke, UK), scrapped, and collected by centrifugation. Proteins were extracted using sample buffer (0.5 M Tris-HCl pH 6.8, 2% SDS, 10% glycerol, 1% β-mercaptoethanol, and 0.03% bromophenol blue) and then processed immediately.

### 4.3. 3D Epidermal Construct Culture, Treatment, and Processing

HaCaT cells were differentiated at the air–liquid interface to generate epidermal equivalents following the method described by Boelsma and colleagues [59], with modifications. Polycarbonate Transwell^®^ membrane inserts with a 0.4 µM pore size (Corning, Corning, NY, USA) were coated with 40 mg·mL^−1^ rat tail type I collagen (Corning) dissolved in 0.1% cold acetic acid and adjusted to ≈pH 7 with 1M NaOH. Transwells were incubated overnight at 5% CO_2_ at 37 °C to allow the collagen to solidify. HaCaT cells (2 × 10^5^ cells·mL^−1^) were seeded onto the coated membranes, then submerged in proliferative media (800 µL) consisting of DMEM/Ham’s F12 media (3:1; Gibco) with 10% FBS (HyClone Laboratories, Logan, UT, USA), 1 µM hydrocortisone and 1.7 µM insulin (Sigma), 50 U·mL^−1^ penicillin–streptomycin and 60 µg·mL^−1^ kanamycin sulphate (Thermo Fisher Scientific), 125 nM transferrin (Sigma), 180 µM adenine (Sigma), 0.84 pg·mL^−1^ cholera toxin (Merck, Rahway, NJ, USA), 10 µM Y27632 (Tocris Bioscience, Bristol, UK), and 15 µM linolenic acid and 7 µM arachidonic acid (Sigma). After 2 days of incubation, the cells were raised to the air–liquid interface and the proliferative media replaced with differentiation media (400 µL) which consisted of proliferation media containing 2.54 mM calcium chloride, 0.57 mM ascorbic acid, and 10 µM rosiglitazone (Sigma) but lacking Y27632. The media were replaced every 2 days for a further 6 days of incubation.

To stimulate epidermal inflammation and barrier dysfunction, 3D constructs were stimulated with TNF-α (20 ng·mL^−1^), IL-4 (50 ng·mL^−1^), and IL-13 (50 ng·mL^−1^). Where indicated, constructs were pre-treated with the extract or vehicle control (1% ethanol) for 2 h, prior to the addition of cytokines. The extract and stimulants were added to the differentiation media below the construct.

For whole-mount immunocytochemistry, constructs were incubated for 24 h, then fixed with ice-cold methanol for 5 min, and processed immediately. For the dye exclusion assay, constructs incubated with cytokines for 24 h were rinsed with PBS, then processed immediately. For chemokine and viability analyses, the conditioned medium was collected by centrifugation after 24 h of incubation, then stored at −80 °C. For Western blot analysis, constructs were incubated for 24 h, rinsed with PBS, scrapped, and then collected by centrifugation. Proteins were extracted using sample buffer and processed immediately.

### 4.4. Cytotoxicity Assays

A crystal violet assay was used to measure the biomass of HaCaT cell monolayers. The medium was removed, then each well was washed with PBS and the cells fixed with ice-cold methanol for 5 min. Then, cells were stained with crystal violet solution (0.25% (*v*/*v*), Sigma) for 10 min at room temperature and rinsed with PBS, and the crystals were solubilised with 33% (*v*/*v*) glacial acetic acid (Merck). Absorbance was read at 595 nm, with percentages of viable cells calculated relative to those of the vehicle-only control.

A CyQuant^TM^ kit (Thermo Fisher Scientific) was used to measure LDH levels in the conditioned media from 3D constructs according to the manufacturer’s instructions. Briefly, sample medium (50 µL) was transferred to a 96-well plate, followed by 50 µL reaction mixture (assay buffer and substrate stock), with incubation for 30 min in the dark. The LDH positive control was also measured. A stop solution (50 µL) was added to all reactions and absorbance was measured at 490 nm and 680 nm (background). The release of LDH was calculated with the formula (E − C)/(T − C) × 100, where E is the absorbance of the conditioned media, C is the absorbance of differentiation media used as the control, and T is the absorbance of the LDH positive control. The percentage of LDH release was normalised to that of cytokine-stimulated cells.

### 4.5. ELISAs

MCP-1 abundance within the conditioned medium from HaCaT cell monolayers and 3D constructs was quantified using a BD-OptEIA™ ELISA kit (San Diego, CA, USA, #2624KI) in accordance with the manufacturer’s instructions. Chemokine abundance was normalised to that of cytokine-stimulated monolayers or constructs.

### 4.6. Immunocytochemistry

Rabbit anti-TNFR1 (ab223352; 1:200, Abcam, Cambridge, UK) and rabbit anti-TRAF2 (ab126758; 1:200, Abcam) were used with Alexa Fluor^®^488 goat anti-rabbit IgG (H + L) and 4′,6-diamidino-2-phenylindole (DAPI; 1:1000, Invitrogen, Carlsbad, CA, USA). Rabbit anti-claudin 1 (ab15098; 1:1000, Abcam) was used with Alexa Fluor^®^594 goat anti-rabbit IgG (H + L) (1:1000, Invitrogen) and DAPI (1:1000). To detect TNFR1 on the cell surface, PFA-fixed HaCaT cell monolayers were incubated with primary antibody for 30 min at 37 °C, followed by secondary antibody for 1 h, then with DAPI for 30 min at room temperature in the dark. To detect intracellular TRAF2 or claudin-1, methanol-fixed HaCaT cell monolayers were permeabilised with 0.1% Triton X-100 (Sigma) and 0.05% Tween 20^®^ (*v*/*v*) (Thermo Fisher Scientific) in PBS for 10 min, then blocked with 1% bovine serum albumin [BSA (*w*/*v*), Gibco Inc.] and 0.1% Tween 20 for 30 min. Cells were incubated with primary antibody overnight at 4 °C and secondary antibody for 1 h, followed by DAPI for 30 min at room temperature in the dark. Coverslips were mounted on glass slides with SlowFade^®^ gold antifade reagent (Invitrogen). Images were captured on the Eclipse No-E upright fluorescence microscope and NIS-Elements C software (Version 3.22, Nikon, Minato City, Japan) for DAPI (excitation 358 nm; emission 461 nm), Alexa Fluor^®^488 (excitation 494 nm; emission 517 nm), and Alexa Fluor^®^594 (excitation 590 nm; emission 617 nm).

### 4.7. Whole-Mount Immunocytochemistry and Confocal Microscopy

Rabbit anti-claudin 1 (ab15098; 1:1000), rabbit anti-keratin-10 (ab76318; 1:100, Abcam), and rabbit anti-filaggrin (ab81468; 1:100, Abcam) were used with Alexa Fluor^®^488 goat anti-rabbit IgG (H + L) or Alexa Fluor^®^594 goat anti-rabbit IgG (H + L) (1:1000, Invitrogen) and 4′,6-diamidino-2-phenylindole (1:1000). Fixed 3D constructs were permeabilised with 0.1% Triton X-100 and 0.05% Tween 20 in PBS for 10 min, then blocked with 1% BSA and 0.1% Tween 20 in 300 mM glycine for 30 min. Constructs were incubated with primary antibody overnight at 4 °C and secondary antibody for 1 h at room temperature, followed by 30 min with DAPI at room temperature in the dark. The cell layers were removed from the Transwell^®^ membrane using a scalpel and mounted on glass slides with SlowFade^®^ gold antifade reagent (Invitrogen). Images were captured on the Nikon Eclipse Ti-E inverted fluorescence microscope for DAPI (excitation 358 nm; emission 461 nm), Alexa Fluor^®^488 (excitation 494 nm; emission 517 nm), and Alexa Fluor^®^594 (excitation 590 nm; emission 617 nm). Multiple Z-stacks were acquired to assess the spatial distribution of cells. Images were processed in NIS-Element C software to generate three-dimensional projections. The two-dimensional images of individual optical slices were combined to obtain 3D montages of Z-stacks.

### 4.8. Western Blotting

Rabbit anti-TNFR1 (ab223352; 1:200), rabbit anti-TRAF2 (ab126758; 1:200), rabbit anti-claudin 1 (ab15098; 1:1000), rabbit anti-GAPDH (ab9485; 1:10,000, Abcam), rabbit anti-β-tubulin (ab6046; 1:10,000, Abcam), and anti-rabbit HRP-conjugated antibody (Invitrogen) were used. Lysates from HaCaT cell monolayers or 3D constructs were boiled, then separated via sodium dodecyl sulphate-polyacrylamide gel electrophoresis using a 12% gel. Proteins were transferred onto a nitrocellulose membrane (0.45 µm; Bio-Rad Laboratories, Hercules, CA, USA), then blocked with 5% (*w*/*v*) non-fat milk in Tris-buffered saline (50 mM Tris, pH 7.6, 150 mM NaCl, and 1% Tween-20) for 1 h at room temperature. Membranes were incubated with primary antibodies in PBS [with 0.05% (*v*/*v*) Tween 20] overnight at 4 °C, secondary antibodies for 1 h at room temperature, and then SuperSignal™ West Pico PLUS substrate (Thermo Fisher Scientific) for 5 min. Signals were visualised using an Alliance gel imaging system (Uvitec, Cambridge, UK). Band densities were quantified using Image J (Version 1.52, National Institutes of Health, Bethesda, MD, USA), then normalised to that of the loading control (β-tubulin) and cytokine-stimulated monolayers or constructs.

### 4.9. Lucifer Yellow Dye Exclusion Assay

An exclusion assay using lucifer yellow dye was used to determine epidermal barrier function following the methods described by [55]. Lucifer yellow (100 µg·mL^−1^, 100 µL, Thermo Fisher Scientific) was administered to the apical side of the cell construct, with PBS (400 µL) added to the basal side. At various time points, half of the PBS (200 µL) was withdrawn from the basal region and replaced with an equivalent volume of PBS. Once the samples had been collected, their relative fluorescence units (RFU) were detected (excitation 485 nm; emission 538 nm) using the SpectraMax Gemini fluorescence plate reader (Molecular Devices, San Jose, CA, USA). The percentage of lucifer yellow leakage was calculated from the RFU of the basal solution relative to that of the RFU positive control (amount of lucifer yellow added to each construct).

### 4.10. GC-MS

The fatty acid methyl esters (FAMEs) were analysed using a Thermo Fisher Scientific TRACE 1300 GC equipped with an ISQ 7000 single quadrupole MS equipped with a Restek Rxi^®^-5Sil MS column. The parameters used for the analysis of FAME were as previously described [44]. The obtained mass spectra were further evaluated by employing the NIST database (MS Search; NIST, MSS Ltd., Manchester, England). The proportion of each fatty acid is reported as a percentage of total integrated fatty acid peak areas for each chromatogram.

### 4.11. Statistical Analyses

Values are expressed as the means ± standard deviation (SD). For the GC-MS analysis, a single *M. pyrifera* extract was assessed in triplicate. For the biological analyses, a single *M. pyrifera* extract was assessed in at least three independent experiments. A Shapiro–Wilk normality test was performed to confirm the normal distribution of the data, followed by one-way analysis of variance (ANOVA) and a post-hoc Dunnett’s test to determine significant points of difference between means. Values of *p* ≤ 0.05 were considered statistically significant.

## Data Availability

All data generated by this study are provided within this article and the Supplementary Materials.

## Supplementary Materials

The following supporting information can be downloaded at: https://www.mdpi.com/article/10.3390/ijms242216383/s1.
