# Supplementary material for: Macrocystis pyrifera Lipids Reduce Cytokine-Induced Pro-Inflammatory Signalling and Barrier Dysfunction in Human Keratinocyte Models
Source: Int J Mol Sci. 2023 Nov 16;24(22):16383. doi: 10.3390/ijms242216383 (PMC10671590; doi:10.3390/ijms242216383)
Supplement: Supplementary file 1 [file ijms-24-16383-s001.zip › ijms-2660215-supplementary.pdf]

International Journal of
Molecular Sciences

MDPI

Supplementary Materials

Macrocystis pyrifera lipids reduce cytokine-stimulated pro-inflammatory signalling and barrier dysfunction in human keratinocyte models

Jamie Mei-Lin Kok [1], Georgina Dowd [2], Jaydee Cabral [3] and Lyn Wise [1,*]

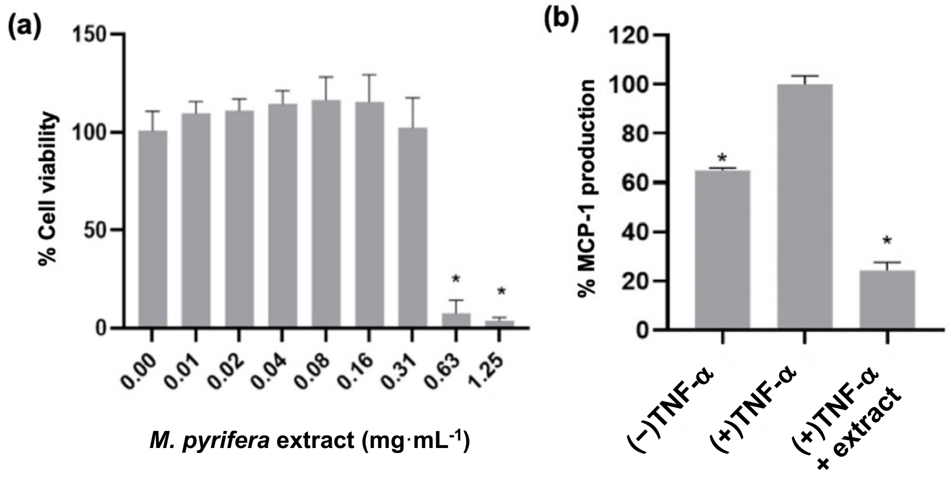

Figure S1. *Macrocystis pyrifera* lipid extract batch #2 suppresses chemokine production in TNF-α-stimulated HaCaT cell monolayers. (**a**) Cells were incubated with varying concentrations of freshly prepared *M. pyrifera* extract with biomass assessed by crystal violet staining after 24 h incubation. Values are expressed relative to the vehicle-only control (0 mg·mL^{-1}). (**b**) Cells were stimulated with TNF-α (20 ng·mL^{-1}) with or without 2 h pre-treatment with extract (0.16 mg·mL^{-1}). After 24 h incubation, the conditioned medium was collected and assayed for MCP-1 production by ELISA. (−) represents unstimulated cells. (+) represents cells stimulated with TNF-α only. Values are expressed as percentages of the (**a**) vehicle or (**b**) (+) controls and presented as means ± SD (n = 3), with those that differ significantly from those controls identified by one-way ANOVA followed by a Dunnett's test (*p ≤ 0.05).

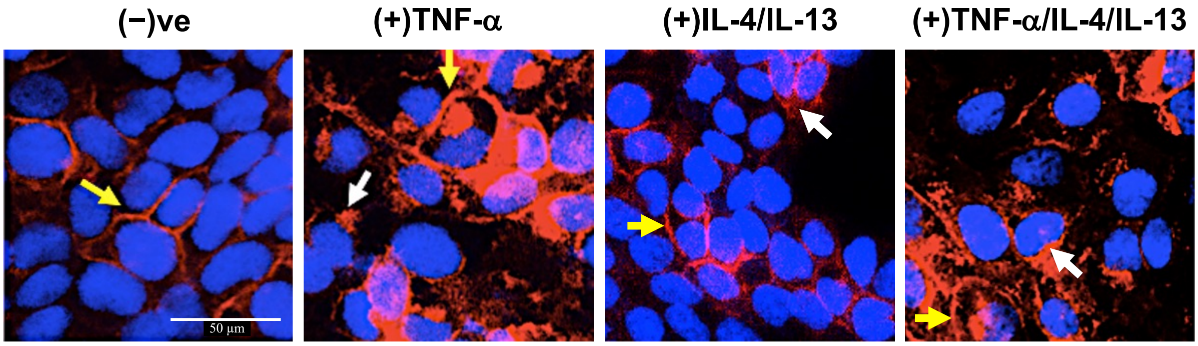

Figure S2. Concurrent stimulation with Th1 and Th2 cytokines produces the greatest disruption to claudin-1 tight junction in HaCaT cell monolayers. Cells were incubated for 24 h with TNF-α (20 ng·mL^{-1}), with or without IL-4 (50 ng·mL^{-1}) and IL-13 (50 ng·mL^{-1}). Cells were fixed then tight junctions stained using rabbit anti-claudin-1 and anti-rabbit Alexa Fluor®594 antibodies (red), with nuclei stained using DAPI (blue). Representative images are shown, with yellow and white arrows indicating intact and disrupted tight junctions, respectively. (−) represents unstimulated cells. (+) represents cells stimulated with cytokines. Scale bar, 50 µm.

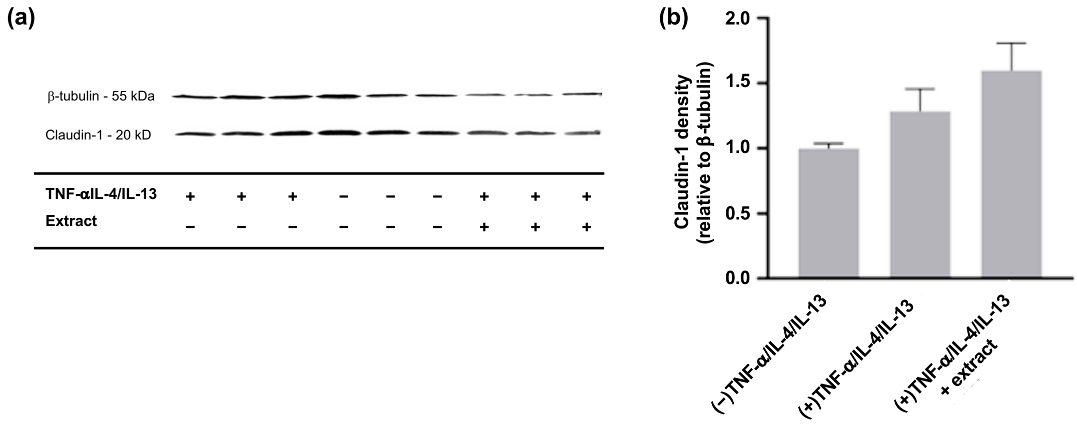

Figure S3. *Macrocystis pyrifera* lipid pre-treatment does not affect claudin-1 protein abundance in cytokine-stimulated HaCaT cell monolayers. Cells were incubated for 24 h with TNF-α (20 ng·mL^{-1}), IL-4 (50 ng·mL^{-1}) and IL-13 (50 ng·mL^{-1}), with or without 2 h pre-treatment with *M. pyrifera* extract (0.16 mg·mL^{-1}). (**a**) Western blot analyses were performed on cell lysates using rabbit anti-claudin-1 and goat-anti-rabbit-HRP antibodies. (**b**) Band densities were determined from (**a**). (−) represents unstimulated cells. (+) represents cells stimulated with cytokines. Values are expressed relative to the (+) control and loading control (β-tubulin) and presented as mean ± SD (n = 3), with those that differ significantly from the (+) control identified by one-way ANOVA followed by a Dunnett's test (*$P \leq 0.05$).

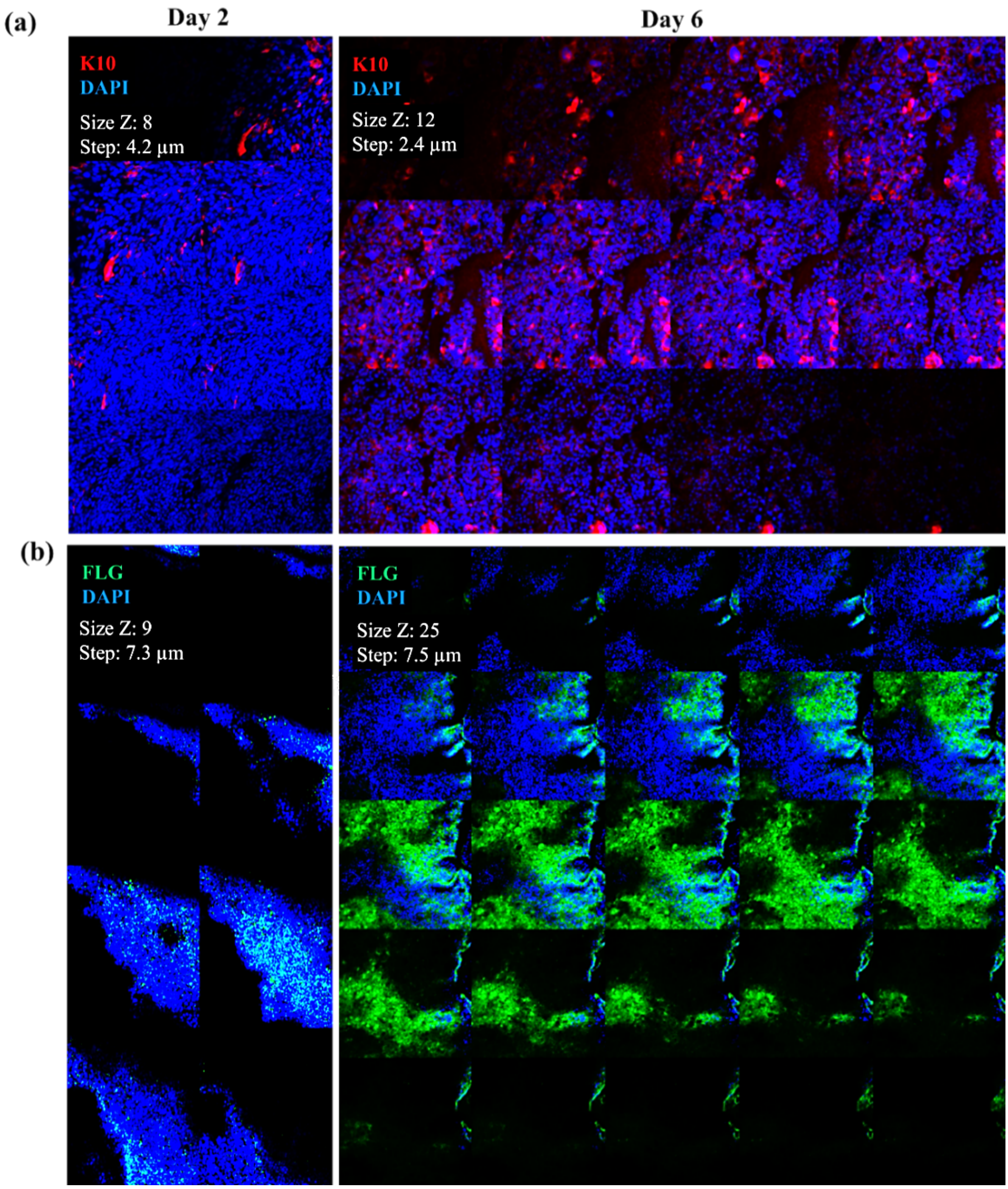

Figure S4. Analysis of cell differentiation within 3D epidermal constructs. HaCaT cells were cultured for 2 days immersed in proliferation media, followed by 6 days of culture at the air liquid interface on differentiation media. At day 2 and 6 of differentiation, the constructs were whole-mount stained with rabbit (**a**) anti-keratin 10 (K10) or (**b**) anti-filaggrin (FLG) and anti-rabbit Alexa Fluor®-594 (red) or Alexa Fluor®-488 (green) antibodies, respectively, and nuclei stained with DAPI (blue). Representative montages of optical slices from confocal Z-stacks of stained cells are shown. The number and depth of slices are indicated.

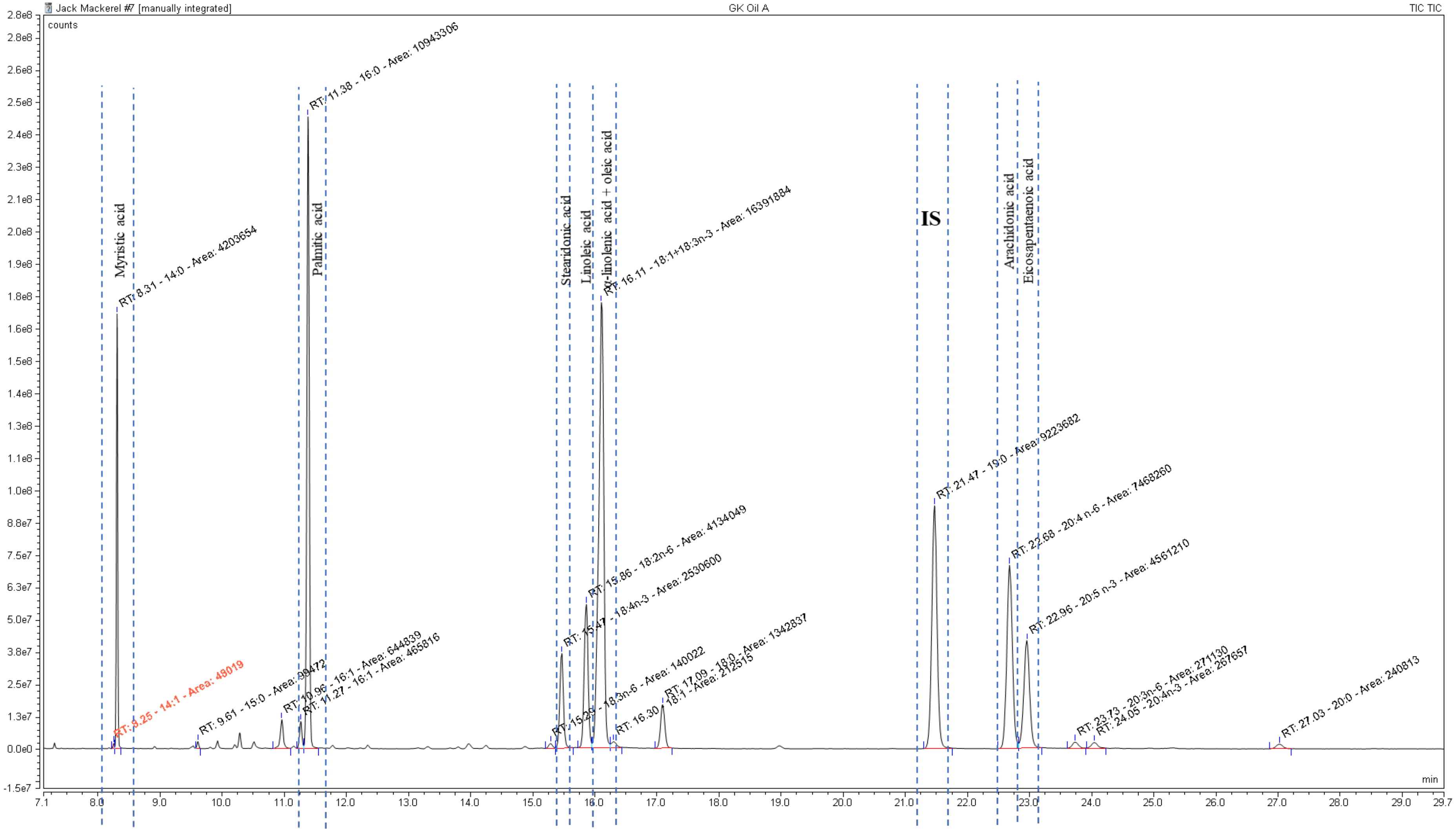

Figure S5. GC chromatogram for the *Macrocystis pyrifera* extract. The peaks for myristic acid (14:0), palmitic acid (16:0), stearidonic acid (18:4n-3), linoleic acid (18:2n-6), co-eluted [oleic acid (18:1) and α-linolenic acid (18:3n-3)], arachidonic acid (20:4n-6) and eicosapentaenoic acid (20:5n-3) are indicated by dotted lines. IS (19:0): internal standard. 18:1 and 18:3n-3 were co-eluted and have similar fragmentation patterns by EI ionisation.

Table S1. Fatty acid composition of *Macrocystis pyrifera* lipid extract batches. Fatty acids were converted to fatty acid methyl esters (FAME) and analysed by gas chromatography-mass spectroscopy.

**Lipid numbers**	**Common name**	**Extract 1**[1]	**Extract 2**[2]
		**% total FAME**[3]	
**Saturated**			
C14:0	Myristic acid	18.7	7.8
C16:0	Palmitic acid	0.2	20.5
C18:0	Stearic acid	1.8	2.5
C19.0	Nonadecyclic acid	15.1	-
**Total**		**36.7**	**31.4**
**Monounsaturated**			
C16:1	Palmitoleic acid	1.3	2.1
C18:1	Oleic acid	16.9	[4]
**Total**		**18.3**	**2.2**
**Polyunsaturated**			
**Omega-6**			
C18:2n-6	Linoleic acid	-	7.7
C20:4n-6	Arachidonic acid	13.5	13.6
**Total**		**14.5**	**22.1**
**Polyunsaturated**			
**Omega-3**			
C18:3n-3	α-linolenic acid	9.9	[4]
C18:4n-3	Stearidonic acid	10.1	4.9
C20:5n-3	Eicosapentaenoic acid	-	8.4
C22:5n-3	Docosapentaenoic acid	10.0	-
**Total**		**30.4**	**13.8**
**Co-elution**			
C18:1+C18:3n-3[4]	Oleic acid / α-linolenic acid		30.7

[1]Previously reported in [44].

[2]Prepared for this study using the Bligh and Dyer method [80].

[3]Lipids representing ≥1% of total FAME in either extract are reported.

[4]18:1 and 18:3n-3 were unable to be quantified owing to co-elution from the column and similar fragmentation patterns by EI ionisation.
